# *Arabidopsis* MYB21 Negatively Regulates *KTN1* to Fine-Tune the Filament Elongation

**DOI:** 10.3390/plants12223884

**Published:** 2023-11-17

**Authors:** Yanan Wang, Lei Shi, Wutao Feng, Ying Fu, Changjiang Li

**Affiliations:** State Key Laboratory of Plant Environmental Resilience, College of Biological Sciences, China Agricultural University, Beijing 100193, China; wangyn620@126.com (Y.W.); 18811065839@163.com (L.S.); 19831126390@163.com (W.F.); yingfu@cau.edu.cn (Y.F.)

**Keywords:** *Arabidopsis*, filament elongation, MYB21, KTN1, transcriptional regulation

## Abstract

The growth process of the stamen filament is crucial for plant reproduction. However, the molecular mechanisms underlying the regulation of filament growth remain largely unclear. Our study has identified that MYB21 is involved in the regulation of filament growth in *Arabidopsis*. In comparison to the wild type, the cell length of the filaments is notably reduced in the *myb21* mutant. Moreover, we found that *KTN1*, which encodes a microtubule-severing enzyme, is significantly upregulated in the *myb21* mutant. Additionally, yeast one-hybrid assays demonstrated that MYB21 can bind to the promoter region of *KTN1*, suggesting that MYB21 might directly regulate the expression of *KTN1*. Finally, transcriptional activity experiments showed that MYB21 is capable of suppressing the driving activity of the *KTN1* promoter. This study indicates that the MYB21-KTN1 module may play a precise regulatory role in the growth of *Arabidopsis* filament cells.

## 1. Introduction

Plant reproductive development is a complex and precisely orchestrated process that plays a pivotal role in the continuation of plant species [1]. One of the vital aspects of sexual reproduction in flowering plants is the development and growth of the stamen. In *Arabidopsis* flowers, there are a total of six stamens, comprising four long ones and two short ones. Each stamen consists of an anther and a stalk-like filament, both highly specialized structures due to their functions. The anther serves as the site of pollen development, while the filament transmits water and nutrients to the anther and positions it to aid in pollen dispersal [2]. Therefore, the male reproductive organs are responsible for plant double fertilization completion, and the successful pollination and subsequent seed formation largely depend on the proper elongation of the stamen filament [1,2,3]. Recent studies have shown that jasmonate (JA), auxin, gibberellin (GA), and brassinosteroid (BR) are involved in the regulation of stamen filament elongation in plants [3,4,5,6,7,8,9,10,11,12,13]. For example, auxin-transport-dependent mechanisms and specific transcription factors are crucial for both the early and late stages of stamen development [4,5]. Additionally, mutants affecting JA biosynthesis, such as *opr3*, and perception, such as *coi1-1*, exhibit a short filament and a male sterility phenotype [6,7]. Moreover, GA triggers DELLA protein degradation, which interacts with JA signaling during stamen development. GA deficiency also results in male sterility due to the over-accumulation of DELLA proteins [6,7,8,9,10,11]. Furthermore, mutants with impaired BR biosynthesis or perception display attenuated stamen filament elongation and a loss of seed production [12,13]. These available data suggest that several transcription factors, such as ARF1/2/6, MYB21/24/57, and BZR1, play very important roles in these hormone pathways to regulate stamen filament elongation [4,5,6,7,8,9,10,11,12,13]. Thus, more work directly focused on stamen growth is necessary to understand the involvement of transcription factors in different processes of stamen development. However, the gene regulatory network mediated by these transcription factors in the filament elongation process is still not clear and requires further investigation.

MYB transcription factors are a family of regulatory proteins that modulate gene expression in response to various developmental and environmental signals [14,15,16,17,18,19]. They have been shown to regulate diverse biological processes in plants, including cell cycle control, hormone signaling, and secondary metabolism [6,7,8,9,10,11,14,15,16,17,18,19]. For instance, MYB3R1 and MYB3R4 are R1R2R3-type MYB transcription factors that regulate the cell cycle and development through the activation of *KNOLLE* transcription in *Arabidopsis* [16]. A R2R3-type MYB transcription factor, PhMYB108, is involved in ethylene and JA-induced petal senescence in *Rosa hybrida* [17]. MYB75 (also known as PRODUCTION OF ANTHOCYANIN PIGMENT1, PAP1) is a R2R3-type MYB transcription factor that plays a key role in regulating the anthocyanin biosynthesis and salt tolerance in *Arabidopsis* [18]. Recent studies also indicated that MYB transcription factors play a crucial role in regulating stamen growth and development. For example, *Arabidopsis* MYB21 and its homologous protein MYB24/57 are reported to play important roles in stamen filament elongation. Among the *myb21* mutant, the *myb21 myb24* double mutant, and the *myb21 myb24 myb57* triple mutant, severe defects in filament elongation leading to male sterility have been observed [3,6,7,8,9,10]. The DELAYED DEHISCENCE1 (DDE1)/OPR3 gene encodes a 12-oxophytodienoic acid reductase3 (OPR3), which was recognized as a function of JA production. Overexpression of *MYB21* or *MYB24* could restore the phenotype of the *opr3* mutant in male sterility [6,8,20]. Cheng et al. found that GA promotes JA biosynthesis to positively regulate the expression of *MYB21*, *MYB24*, and *MYB57*, which further promotes the elongation of stamen filament cells in *Arabidopsis* [6]. Furthermore, Huang et al. found that DELLAs could also directly interact with MYB21 or MYB24 to regulate stamen filament elongation by inhibiting the transcriptional function of MYB21 and MYB24, which also indicates that GA and JA can cross-talk to regulate stamen filament elongation by targeting MYB21 and MYB24 in *Arabidopsis* [8,10]. However, the downstream factors of MYB21 or MYB24 in the filament elongation of plants are still largely unclear.

The plant cytoskeleton, capable of offering mechanical support to both the cell and its cytoplasmic constituents, plays a pivotal role in numerous cellular processes that are essential for cell morphogenesis and organogenesis [21]. Microtubule (MT) is a critical constituent of the cellular cytoskeleton, and research has increasingly demonstrated a close relationship between the establishment of cell morphology in plant cells and the cortical MT array and dynamics within the cell [21,22]. *KTN1*, which encodes a katanin p60 subunit, plays an important role in plant cell morphogenesis [12,22,23,24,25]. Studies have shown that KTN1 and KTN80 form KTN1-KTN80 heterodimers, which was activated by BZR1-family transcription factors (BFTFs), thereby associated with BRs signaling pathway to precisely perform MT severing and properly regulate filament elongation in *Arabidopsis* [12,22]. Another study indicated that the ROP6-RIC1 module could activate KTN1 to sever cortical MTs in *Arabidopsis* cells [24]. KTN1 loss-of-function mutants show multiple morphogenesis phenotypes in *Arabidopsis*, such as short hypocotyl, inflorescence stems having short internodes, and reduced stamen length [12,22,23,24,25]. Recently, a study has shown that BR signaling positively regulates the expression of *KTN1* in *Arabidopsis* stamens. The core transcription factor in BR signaling, BZR1, can directly bind to the promoter of *KTN1* and activate the expression of *KTN1* to maintain stamen filament cell elongation [12]. Interestingly, the overexpression of *KTN1* in *Arabidopsis* also causes the short filament phenotype [12]; this indicates that the *KTN1* expression level must be precisely controlled in plants. However, which TF(s) negatively regulates the expression of *KTN1* remains unclear.

In this study, we evaluated MYB21’s role in *Arabidopsis* filament elongation and confirmed its importance using the *myb21* mutant. We also identified MYB21 as a potential negative regulator of *KTN1* expression. Together with previous research, our findings suggest that the MYB21-KTN1 module plays a vital role in precise stamen filament elongation in *Arabidopsis*.

## 2. Results

### 2.1. MYB21 Positively Regulates the Stamen Filament Elongation in Arabidopsis

**Figure 1:**
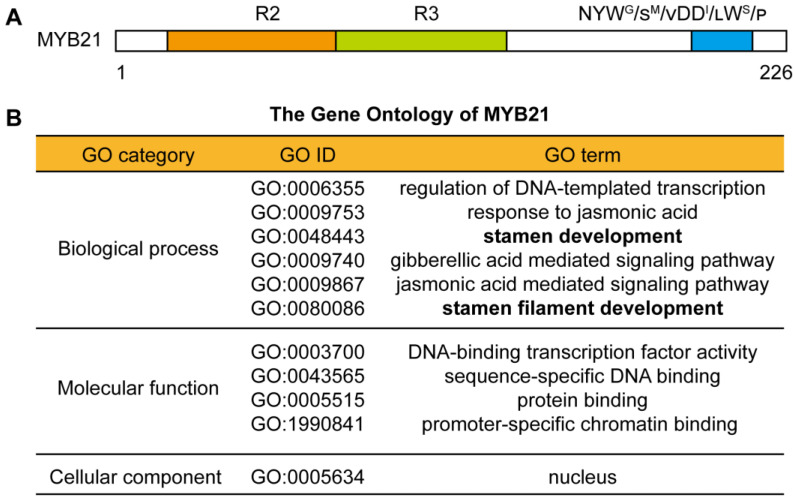
MYB21 is an R2R3-type MYB transcription factor regulating stamen filament development in *Arabidopsis*. (**A**) The primary structure of MYB21 protein shows a conserved R2R3 domain in the N terminus and an NYW^G^/_S_^M^/_V_DD^I^/_L_W^S^/_P_ domain in the C terminus, which are highlighted in orange, green, and blue, respectively. (**B**) The Gene Ontology (GO) analysis of MYB21 shows that MYB21 is involved in biological processes such as “stamen development” and “stamen filament development”. The data were obtained by using the PlantPAN4.0 tool (http://plantpan.itps.ncku.edu.tw/plantpan4/index.html) accessed on 8 October 2023.

In order to analyze the molecular mechanism of MYB21-mediated stamen filament elongation, we analyzed the primary structure of MYB21 by using the SMART tool [26]. MYB21 consists of a DNA-binding domain (R2R3 domain) located at the N terminus and a regulatory region located at the C terminus (including a NYW^G^/_S_^M^/_V_DD^I^/_L_W^S^/_P_ motif) (Figure 1A). This result is similar to previous reports [6,7], which indicate that MYB21 is a R2R3-type MYB transcription factor. In addition, the GO analysis performed by the PlantPAN4.0 tool revealed that MYB21 is involved in biological processes, such as the “regulation of DNA-templated transcription”, the “response to jasmonic acid”, “stamen development”, and “stamen filament development” (Figure 1B), which was also verified by previous studies [6,7,8,9]. The GO category of cellular components indicates that MYB21 functions in the nucleus, which is also consistent with its function as a transcription factor (Figure 1B).

**Figure 2:**
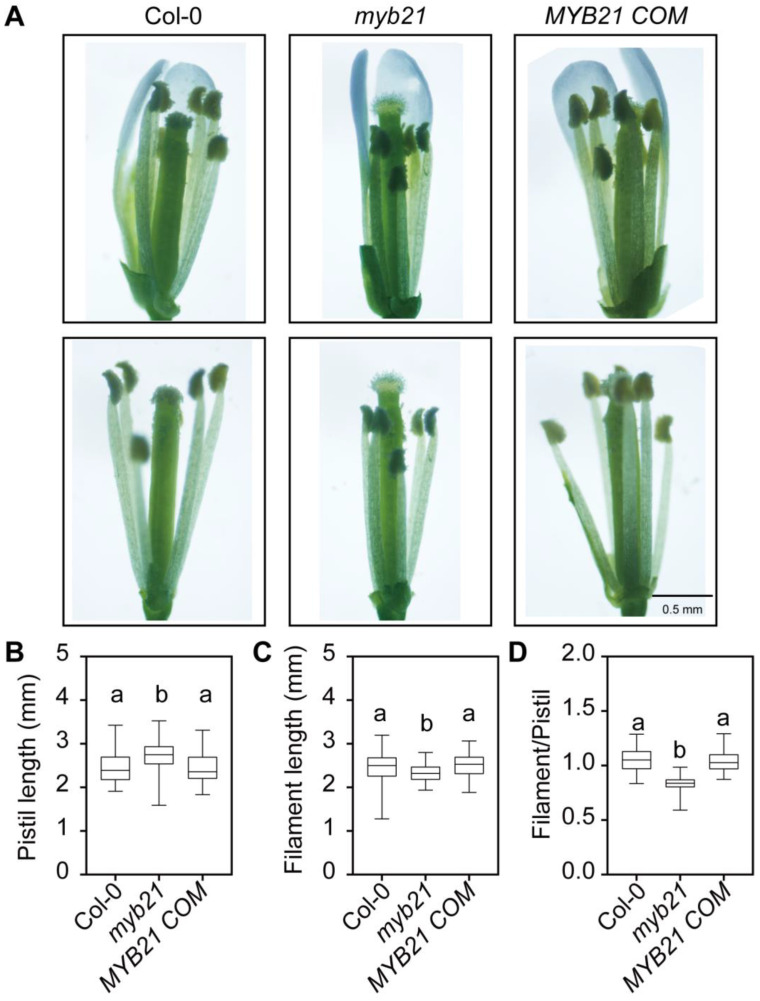
Comparison of flowers at floral stage 13 in Col-0, *myb21*, and *MYB21 COM* line. (**A**) Floral organs develop uncoordinatedly in the *myb21* mutant, the papillae of the *myb21* mutant stigmas are longer than Col-0, and the anther filament lengths are shorter than Col-0. The phenotypes of the *MYB21 COM* line are similar to Col-0. The upper panel shows flowers with sepals and petals before removal, while the lower panel shows petals removed. Scale bar = 0.5 mm. (**B**–**D**) Statistical analysis of pistil length, filament length, and the ratio of filament length and pistil length in Col-0, *myb21*, and *MYB21 COM* line shown in A. The data in (**B**–**D**) are presented as mean ± SD (*n* ≥ 20); statistical significance was determined by one-way ANOVA. Significant differences at *p* < 0.05 are indicated by different lowercase letters. The boxplots reflect 25%, 50%, and 75% and the maximum/minimum of the total value.

To further identify the molecular regulatory network of MYB21-mediated stamen filament elongation, we reevaluated MYB21’s function during stamen filament development. We observed stage 13 of the flora organs of both the wild type (Col-0) and the *myb21* single mutant; as shown in Figure 2, the lengths of the pistils in the *myb21* mutant are longer than those in the wild type, while the stamen filaments length of *myb21* mutant are shorter than wild type (Figure 2A–C). This results in the uncoordinated growth of both the stamens and pistils in the *myb21* mutant (Figure 2A,D). Moreover, the phenotype of *myb21* could be rescued in the *MYB21* complementation line (*MYB21 COM*) (Figure 2A–D). This confirms that MYB21 is indeed responsible for the phenotype of the *myb21* single mutant. Additionally, our results are consistent with previous reports [6,7,8,9], all of which indicate that MYB21 plays a positive role in regulating stamen filament elongation in *Arabidopsis*.

### 2.2. MYB21 Positively Regulates Filament Cell Elongation in Arabidopsis

**Figure 3:**
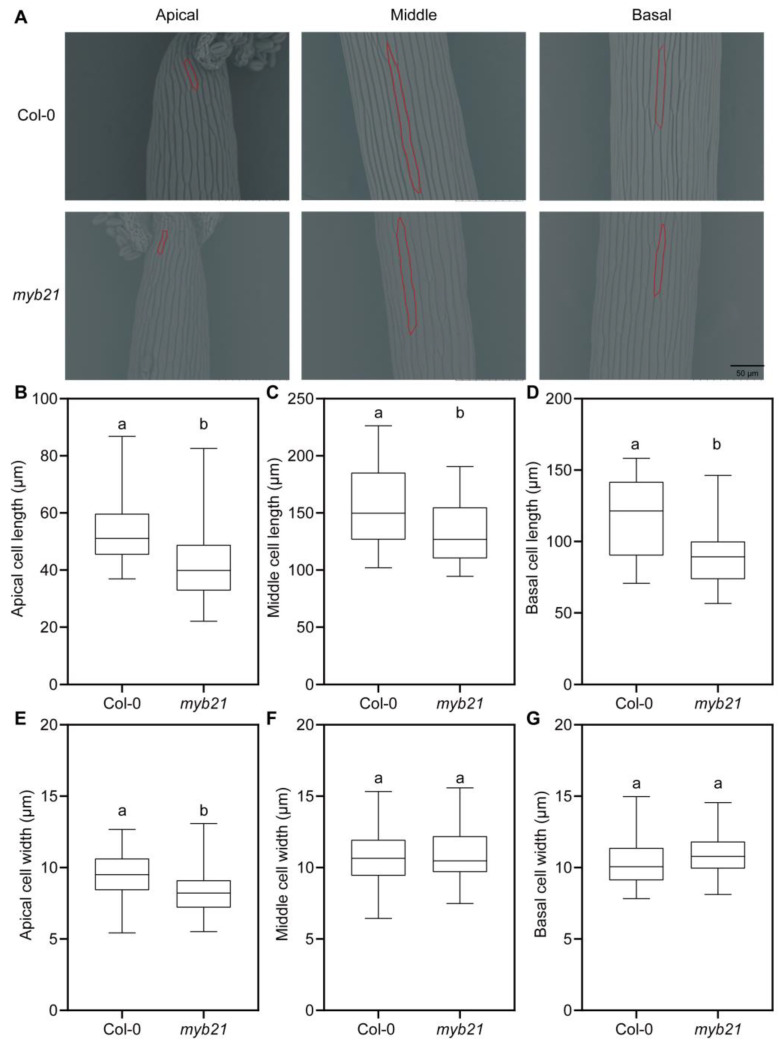
MYB21 controls stamen filament elongation. (**A**) Filament epidermal cells at stage 13 from Col-0 and *myb21* mutant were observed by scanning electron microscopy (SEM). Segments are shown here from the apical, middle, and basal parts of the filament, respectively. The individual cell is outlined with a red line for visualization. The *myb21* mutant appeared shorter than Col-0. Scale bar = 50 µm. (**B**–**G**) Quantification of filament cell length and width in panel (**A**). The data in (**B**–**G**) are presented as mean ± SD (*n* ≥ 20); statistical significance was determined by one-way ANOVA. Significant differences at *p* < 0.05 are indicated by different lowercase letters. The boxplots reflect 25%, 50%, and 75% and the maximum/minimum of the total value.

To further investigate whether MYB21 regulates the elongation of filament cells, we measured cell lengths and widths at the apical, middle, and basal regions of wild-type and *myb21* mutant filaments. The results revealed that the lengths of the filament cells at the apical, middle, and basal regions in *myb21* mutants are all significantly shorter than those in the wild type (Figure 3A–D). As for cell widths, we observed that only the apical part of *myb21* mutants is narrower than in the wild type, with no significant differences in the middle and basal regions of the filaments between the *myb21* mutant and the wild type (Figure 3A,E–G).

Organ development is intricately influenced by patterns of cell division and elongation. To better understand the cellular basis of reduced filament elongation and to assess additional growth parameters, we counted the number of cells in the filament epidermis via microscopy and examined the stamen count at flowering stage 13 [27]. When compared to Col-0, the *myb21* mutant and the *MYB21 COM* line exhibited similar cell numbers and stamen counts (Supplemental Figure S1). This suggests that the reduction in filament length in the *myb21* mutant is attributed to a defect in cell elongation rather than cell division and the initiation of stamen development. Additionally, we assessed vascularization in the filaments at stage 13 through resin-embedded cross-sections [28], and no significant differences were observed in the vascularization of Col-0, *myb21*, and the *MYB21 COM* line (Supplemental Figure S2). These results indicate that *MYB21* regulates filament cell elongation but not the early development of stamen filaments. Furthermore, pollen viability is a significant factor determining male infertility. We examined pollen viability in the anthers of Col-0, *myb21*, and the *MYB21 COM* line using Alexander staining; as shown in Supplementary Figure S3, the pollen grains in the *myb21* single mutant anthers were found to be viable. In summary, our findings suggest that MYB21 primarily regulates the length of filament cells, thereby influencing the elongation process of the filaments.

### 2.3. MYB21 Negatively Regulates the Expression of KTN1

**Figure 4:**
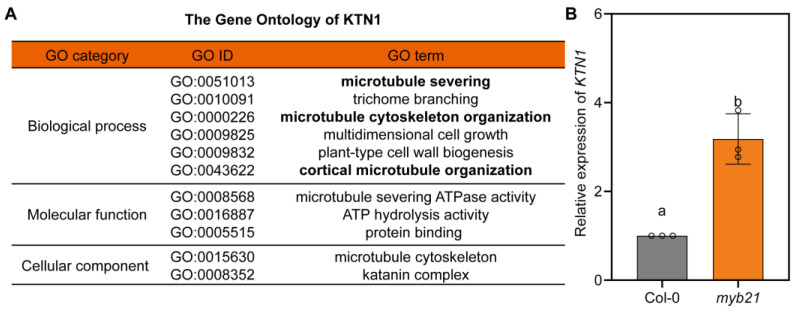
MYB21 negatively regulates the expression of *KTN1*. (**A**) The GO analysis of KTN1 shows that KTN1 is involved in biological processes such as “microtubule severing”, “microtubule cytoskeleton organization”, and “cortical microtubule organization”. The data were obtained by using the PlantPAN4.0 tool. (**B**) Relative expression of *KTN1* in the stage 13 flowers of the Col-0 and *myb21* mutant, which was determined by RT-qPCR. The dots on the bar chart represent the original data points. Statistical significance was determined by one-way ANOVA. Significant differences at *p* < 0.05 are represented by different lowercase letters.

Previous studies indicated that KTN1 is a key downstream factor of the BZR1 family transcription factors involved in maintaining filament morphogenesis in *Arabidopsis* [12]. We then performed a GO analysis of KTN1 using the PlantPAN4.0 tool, and the results shown in Figure 4A indicate that KTN1 is involved in biological processes such as “microtubule severing”, “microtubule cytoskeleton organization”, and “cortical microtubule organization”. Together with previous studies [12,22,23,24,25], these analyses all indicate that KTN1 could regulate the cell morphogenesis of stamen filament cells. We wondered whether MYB21 could also regulate the expression of *KTN1* in *Arabidopsis*. To investigate this, we conducted an RT-qPCR assay. Stage 13 floral organs from the wild type and the *myb21* mutant were collected, and total RNA was extracted and reverse transcribed for the RT-qPCR assay. Surprisingly, the results showed that the expression of *KTN1* in *myb21* mutants was significantly upregulated compared to the wild type (Figure 4B). This suggests that, unlike BZR1, MYB21 may negatively regulate the expression of *KTN1* in *Arabidopsis* filaments.

### 2.4. MYB21 May Directly Bind to the Promoter of KTN1

**Figure 5:**
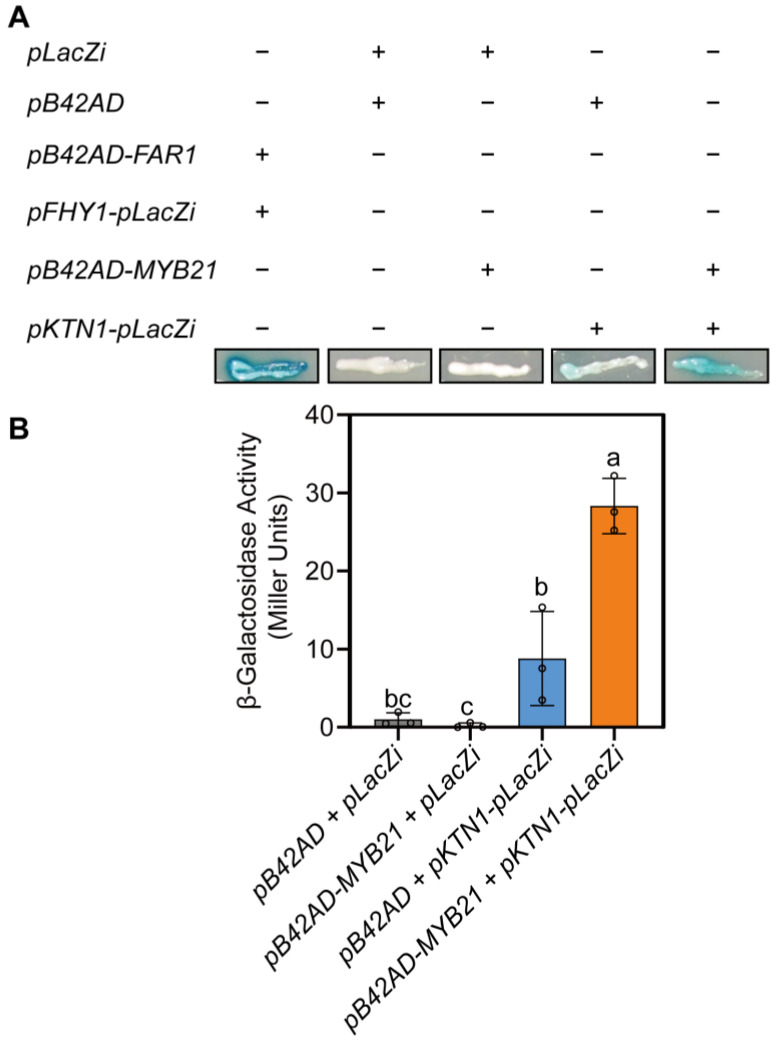
MYB21 may directly bind to the promoter of *KTN1*. (**A**) Transcription factor activity of MYB21 verified by yeast one-hybrid analysis; *pB42AD-MYB21* and *pKTN1-pLacZi* were co-transformed into yeast strain *EGY48*; the *pFHY1-pLacZi* and *pB42AD-FAR1* combination was used as a positive control; *pB42AD* and *pLacZi*, *pB42AD-MYB21* and *pLacZi*, and *pB42AD* and *pKTN1-pLacZi* were used as negative controls. The clones harboring *pB42AD-MYB21* and *pKTN1-pLacZi* induced LacZ activity and turned blue, indicating that MYB21 can bind to the *KTN1* promoter. (**B**) Liquid culture assays showing expression of the *LacZ* reporter gene; β-galactosidase activities were measured using ONPG as substrate. The dots on the bar chart represent the original data points. Error bars indicate SD of three independent yeast clones. Different letters represent significant differences at *p* < 0.05 by one-way ANOVA.

To determine whether MYB21 directly targets *KTN1*, we conducted a yeast one-hybrid assay. We obtained the 2.0 kb promoter region of *KTN1* (upstream of the ATG start codon) through PCR and then cloned it into the *pLacZi* vector as a reporter. *MYB21* was cloned into the *pB42AD* vector as an effector. After co-transforming the *pB42AD-MYB21* and *pKTN1-pLacZi* vectors into the yeast strain *EGY48*, we assessed the potential binding of MYB21 to the *KTN1* promoter by observing a blue color change in yeast cells in the presence of X-gal. The results revealed that the *KTN1* promoter within the *pLacZi* vector exhibited some degree of self-activation in yeast. Notably, the *pB42AD-MYB21*/*pKTN1-pLacZi* transforming pair exhibited a deeper blue color than the *pB42AD*/*pKTN1-pLacZi* transforming pair (as shown in Figure 5A). This observation suggests that MYB21 may directly bind to the *KTN1* promoter.

Furthermore, to investigate the binding effect of MYB21 on the *KTN1* promoter, we conducted a yeast liquid culture assay in which we used ONPG as a substrate to examine the activity of β-galactosidase. The results depicted in Figure 5B demonstrate that the coexpression of *pKTN1-pLacZi* and *pB42AD-MYB21* resulted in higher β-galactosidase activity compared to the coexpression of *pKTN1-pLacZi* and *pB42AD* (control). This finding provides additional evidence supporting the notion that MYB21 likely binds directly to the *KTN1* promoter.

### 2.5. MYB21 Represses the Activity of the KTN1 Promoter

**Figure 6:**
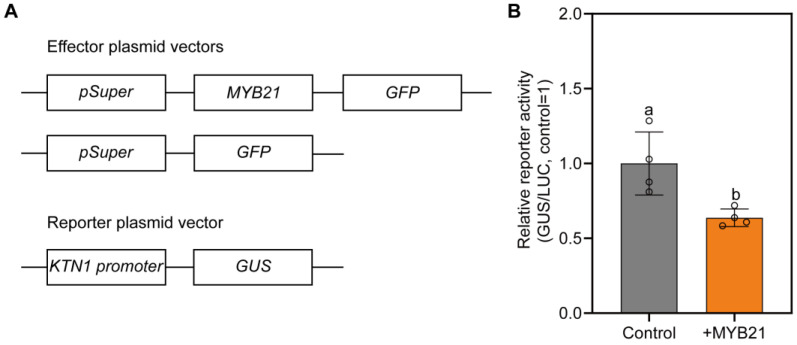
MYB21 repressed the activity of the *KTN1* promoter. (**A**) Schematic diagram of the effector and reporter constructs for the transient expression assays in *Nicotiana benthamiana*. (**B**) Transient transactivation assay of MYB21 with the *KTN1* promoter. The *pSuper::LUC* plasmid was the internal control. The dots on the bar chart represent the original data points. Error bars indicate the SD of four independent assays. A letter represents a significant difference at *p* < 0.05 by one-way ANOVA.

MYB21 was involved in regulating stamen filament elongation, and it may have a negative role in *KTN1* expression. To investigate whether MYB21 could repress *KTN1* expression in vivo, transactivation assays were performed in tobacco leaves. The *GUS* reporter gene was driven by the 2.0 kb promoter of *KTN1* (upstream of the ATG start codon), and the sequence of *MYB21* was fused to a *pCAMBIA1300* vector driven by the *Super* promoter, which served as an effector to evaluate whether it was targeting the reporter gene promoter (Figure 6A). As revealed in Figure 6B, the co-transfection of the *pKTN1::GUS* effector and the control (*pSuper::GFP*) resulted in the activation of the *GUS* reporter gene. However, the co-transfection of *pSuper::MYB21-GFP* and *pKTN1::GUS* led to a significant decrease in GUS activity, demonstrating a role for MYB21 in the repression of *GUS* reporter gene expression (Figure 6B) and indicating that MYB21 may repress *KTN1* expression in plants.

## 3. Discussion

**Figure 7:**
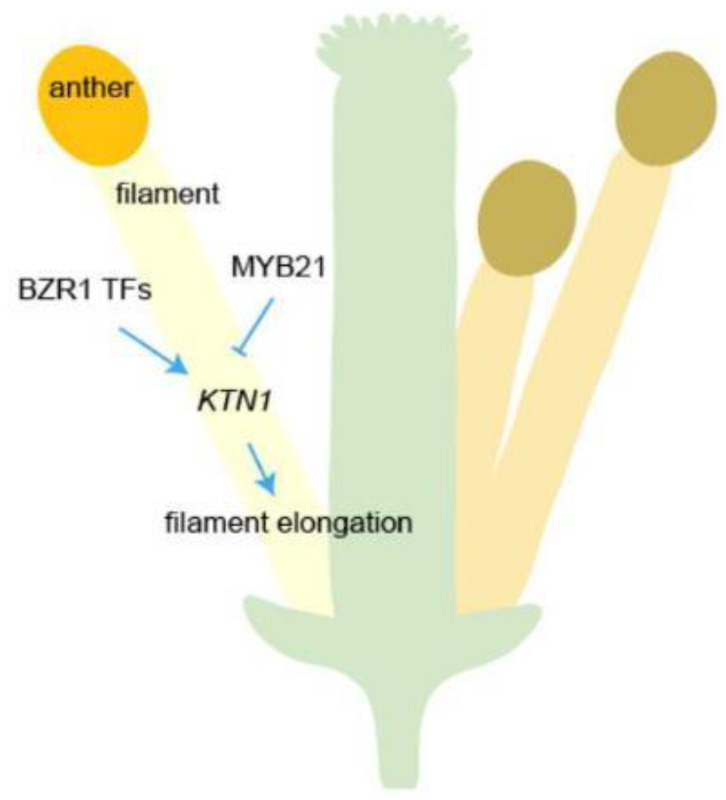
A simplified model of MYB21-KTN1 regulation in *Arabidopsis* filament elongation, based on [12,13], illustrates that the transcription factors MYB21negatively regulate the expression of *KTN1* to ensure the accurate elongation of stamen filaments.

Despite the growth of stamen filaments in plants is a pivotal process for successful reproduction, the intricate molecular mechanisms orchestrating stamen filament growth remain largely enigmatic. In this report, we identified that the MYB21-KTN1 module may play an important role in precisely maintaining filament elongation in plants. As shown in Figure 7, KTN1 may act as a downstream factor of MYB21 to regulate the morphogenesis of filaments. BZR1 family transcription factors can positively regulate the expression of *KTN1* [12], which implies that BZR1 and MYB21 may antagonistically and finely regulate the expression of *KTN1* to keep the filament cell properly elongated (Figure 7).

In every stage of stamen development, plant hormones are extensively involved. Previous studies have shown that MYB21 plays a role in the JA and GA signaling pathways [6,7,8,9,10,11], but whether the MYB21-KTN1 module is regulated by JA or GA still needs further investigation. One intriguing avenue for future research is to explore the potential crosstalk between the MYB21-KTN1 module and other regulatory pathways known to influence filament growth. For instance, BR-BZR1 signaling positively regulates *KTN1* expression [12]. This raises the question of whether the JA-MYB21 module and the BR-BZR1 module engage in antagonistic interactions to finely tune *KTN1* expression levels. Investigating the interplay between MYB21 and BZR1, as well as the possibility of the involvement of other transcription factors or signaling molecules, could provide a more comprehensive view of the regulatory network governing stamen filament elongation.

Another study has shown that KTN1 is involved in ABA-signaling-mediated root hydrotropism [25] and is also implicated in auxin/BR-signaling-mediated cell morphogenesis in *Arabidopsis* [12,25]. These findings suggest that KTN1 serves as a key node in mediating various plant hormones to regulate plant development and growth processes. It would be intriguing to further investigate whether the ABA-KTN1 module plays a role in plant stamen filament development in the future.

In recent years, an increasing number of R2R3-MYB transcriptional repressors that participate in the regulation of various aspects of plant growth and development. For example, *Arabidopsis* MYB32 is involved in regulating lignin biosynthesis and pollen development, while MYBL2 is involved in suppressing the anthocyanin biosynthesis process [29,30]. In this study, we have discovered that MYB21 plays a negative regulatory role in *KTN1* expression. Moreover, there is evidence suggesting that MYB21 might directly bind to the *KTN1* promoter and inhibit its activity. A previous study has shown that MYB21 can act as a transcriptional activator [10]; that is to say, MYB21 may have dual functions in transcriptional activation and transcriptional repression, similar to the known transcription factor BES1 in *Arabidopsis* [13,30]. Traditional transcriptional repressors typically feature an ERF-associated amphiphilic repression (EAR) domain (LxLxLx or DLNxxP) [29,30,31,32,33]. However, it is worth mentioning that we did not identify an EAR domain within MYB21. This suggests the possibility that MYB21 may contain an as-yet-undiscovered transcriptional repression domain, or alternatively, that MYB21 could recruit a repressor to downregulate *KTN1* expression. Further exploration is needed to elucidate the precise molecular mechanism through which MYB21 suppresses *KTN1* expression.

The R2R3-MYB family (R2R3-MYBs) constitutes one of the largest transcription factor gene families in plants, exhibiting significant structural and functional diversity [14,15]. Some R2R3-MYBs are involved in floral organ development and maturation in plants. For instance, the R2R3-MYB transcription factor EMISSION OF BENZENOIDS II (EOB2) is known to play a role in flower bud senescence and petal/pistil maturation in Petunia axillaris [34]. More research on R2R3-MYB transcription factors will help us better understand the intricate transcriptional regulation network during flower development and organ identity. Interestingly, MYB24 and MYB57 are proteins that are homologous to MYB21, and all three are key transcription factors in the JA signaling pathway [6,7,8,9,10,11]. Whether MYB24 and MYB57 also have the potential to regulate *KTN1* expression through JA signaling remains a topic requiring future investigation.

In summary, filaments are morphologically critical components of the stamen with a centrally located vascular bundle, and the roles of the stamen include not only the transport of water and nutrients but also the provision of mechanical support for the anthers. Delving more deeply into the relationships between the MYB21-KTN1 module and other regulatory pathways promises to unravel a more comprehensive and interconnected network governing stamen filament elongation in plants. Such insights may not only advance our understanding of fundamental plant biology but also hold implications for potential applications in agriculture and crop improvement.

## 4. Materials and Methods

### 4.1. Plant Materials and Growth Conditions

*Arabidopsis thaliana* ecotype Col-0 was used as the wild type in this study. The *myb21* (SALK_042711) was obtained from the *Arabidopsis* Biological Resource Center (ABRC). The homozygous lines were identified using a PCR-based approach. The transgenic homozygous *Arabidopsis* line of the T3 generation (*MYB21 COM*) was used in this study. Total RNA from stage 13 flora organs was extracted according to the manufacturer’s instructions using an RNAprep Pure Plant Kit (S8114; Tiangen, Beijing, China). The ChamQ Universal SYBR qPCR Master Mix (Q711-02; Vazyme Biotech Co. Ltd., Nanjing, China) was used for amplification. The primers are listed in Supporting Information Table S1.

Seeds were sterilized in 5% (*v*/*v*) sodium hypochlorite for 15 min, subsequently washed with sterilized water four times, and treated in the growth medium at 4 °C in the dark for 3 d. The growth medium contained half-strength Murashige and Skoog medium (1/2 MS) with 0.8% plant agar (PhytoTechnology Laboratories, Shawnee Mission, KS, USA). Young seedlings were grown on 1/2 MS culture plates in a growth chamber at 22 °C with a 16/8 h light/dark photoperiod before being transferred to the soil. *Arabidopsis* adult plants were grown in the greenhouse with a 16/8 h light/dark photoperiod and a light density of ~120 µmol m^−^^2^ s^−^^1^ at 22 °C until flowering.

### 4.2. Plasmid Construction and Generation of Transgenic Arabidopsis Plants

The gDNA was extracted using the CTAB method. A 2.0 kb region of the *KTN1* promoter (upstream of the ATG start codon) was amplified and inserted into the pCAMBIA1391 vector to prepare the *pKTN1::GUS* construct. *pSuper::MYB21-GFP* was constructed for the GUS/LUC assay. The cDNA sequences of *MYB21* were obtained from the *Arabidopsis* Information Resource (http://www.arabidopsis.org) accessed on 14 November 2018. Then, the sequence of *pMYB21::MYB21-GFP* was colned into the plant transformation vector *pCAMBIA1300* and transformed into *myb21* mutant plants to produce the complemented line. Plants were transformed using *Agrobacterium tumefaciens* strain *GV3101* using the floral dip method [35]. The specific primers were designed using Primer5.0, synthesized at BGI Genomics (Shenzhen, China), and used to amplify the coding sequence, which is listed in Table S1.

### 4.3. Yeast-One-Hybrid (Y1H) Assay

The coding sequence of *MYB21* was cloned into a pB42AD vector, and a 2.0 kb region of *KTN1* promoter was cloned separately into the pLacZi vector. The respective combinations of plasmids were co-transformed into yeast-competent cells (*EGY48*; Huayueyang Biotech Co. Ltd., Beijing, China). Yeast transformation was performed as described previously [36]. The transformed yeast cell suspension was transferred to SD-Trp-Ura medium containing 0.04 g/L 5-bromo-4-chloro-3-indolyl-β-D-galactopyranoside (X-Gal), 1× BU salts (9.35 g/L Na_2_HPO_4_·12H_2_O, 3.9 g/L NaH_2_PO_4_·H_2_O, pH 7.0) and incubated at 30 °C to observe blue color changes.

The liquid culture assays were designed to quantify the activity of β-galactosidase, which is encoded by the *LacZ* gene under the control of the *KTN1* promoter. This assay reflects the binding effect of MYB21 on the *KTN1* promoter in the reconstituted yeast system. The method is described in a previous report [37]. In detail, o-nitrophenol-β-D-galactopyranoside (ONPG) was used as the substrate for quantitative assays. The transformants were cultured in a liquid medium at 30 °C with shaking at 200 rpm. Yeast concentration was estimated by measuring the culture’s absorbance at 600 nm. To prepare the cell mixtures for analysis, we used a Z buffer (containing 21.5 g/L Na_2_HPO_4_·12H_2_O, 6.2 g/L NaH_2_PO_4_·2H_2_O, 0.75 g/L KCl, 0.246 g/L MgSO_4_·7H_2_O, pH 7.0) to resuspend the yeast culture. The cell mixtures were then harvested by centrifugation. To initiate the assay, we added chloroform and 0.1% SDS to the Z buffer, which also contained 0.27% (*v*/*v*) β-mercaptoethanol. The mixture was vortexed to ensure thorough mixing of the cell pellet. Next, we immediately added ONPG (dissolved in Z buffer) to initiate the enzymatic reaction. The reaction was considered complete when a yellow color developed in the reaction buffer, at which point we added Na_2_CO_3_ to stop the reaction and recorded the time. The β-galactosidase units were calculated as follows:

Miller Units = 1000 × A420/(OD600 × culture volume [mL] × reaction time [min])

### 4.4. Reverse-Transcription Quantitative PCR (RT-qPCR) Analysis

Total RNA was extracted from the *Arabidopsis* flora organs using an RNA extraction kit (DP432; TIANGEN, Beijing, China). Subsequently, RNA was reverse transcribed using SuperScript III reverse transcriptase (18080044, Thermo Scientific, Waltham, MA, USA) to obtain the strand cDNA. Quantitative real-time PCR analysis was performed using the ABi7500 real-time PCR system with the ChamQ Universal SYBR qPCR Master Mix (Q711-02; Vazyme Biotech Co. Ltd., Nanjing, China), and *EF1-α* was used as an internal control. The procedure of RT-qPCR consisted of a two-step temperature cycle with pre-degeneration at 95 °C for 30 s, 39 cycles of degeneration at 95 °C for 5 s, and an extension step at 58 °C for 30 s. The RT-qPCR experiment was repeated for three independent biological replicates. The sequences of primers used for RT-qPCR are listed in Table S1.

### 4.5. Light Microscopy and Scanning Electron Microscopy

To analyze the phenotype of *myb21*, the pistil and longest filament of flowers at floral stage 13 were collected and observed under an Olympus SZX16 microscope (Tokyo, Japan). The flower stages were defined as reported previously [38]. For scanning electron microscopic examination of filament cells, fresh anther filaments were spread onto the surface of adhesive tape pieces and observed using a scanning electron microscope (TM3000, Hitachi, Tokyo, Japan) at an accelerating voltage of 15 kV. The experiment was repeated for three independent biological replicates. The lengths and widths were measured using ImageJ software (National Institutes of Health, http://rsb.info.nih.gov/ij; v.1.38) accessed on 21 August 2023.

### 4.6. Transient Expression Assays in Nicotiana benthamiana

The transient *GUS* expression assays were performed as described [39]. The constructs *pKTN1::GUS* and *pSuper::MYB21-GFP* were transformed into *Agrobacterium* (strain GV3101) separately. For every infiltration sample, *pSuper::Luciferase (LUC)* was added as an internal control. Agrobacterium cells were harvested by centrifugation and suspended in a solution containing 10 mM MES, pH 5.6, 10 mM MgCl_2_, and 200 mM acetosyringone at an optical density (600 nm) of 0.7, incubated at room temperature for 4 h, and then used to infiltrate leaves of *N. benthamiana* using a needle-free syringe. The infiltrated plants were kept under a 14 h light/10 h dark photoperiod at 22 °C for 72 h to express GUS and Luciferase (LUC) proteins. The F4500 fluorescence spectrophotometer (Hitachi, Tokyo, Japan) was used to measure GUS activity, and 4-methylumbelliferyl-b-D-glucuronide (MUG) was used as a substrate in this fluorometric assay. LUC activities were measured using a Promega GLOMAX 20/20 luminometer, GUS activities were normalized to the LUC activities. Then the GUS/LUC ratios were calculated, and these data were used to quantify the promoter activity.

### 4.7. Bioinformatic Analysis

For the bioinformatic analysis of MYB21 or KTN1, the SMART tool (http://smart.embl-heidelberg.de/, accessed on 8 October 2023) was used to analyze the primary structure of MYB21 [26]; there was no known EAR motif detected in the MYB21 protein. PlantPAN4.0 (http://plantpan.itps.ncku.edu.tw/plantpan4/index.html, accessed on 8 October 2023) was used to analyze the Gene Ontology of MYB21 and KTN1 [40].

### 4.8. Alexander Staining Assay

Pollen viability plays a crucial role in plant double fertilization, successful fertilization is influenced by the percentage of viable pollen grains relative to the total pollen grains in an anther [26]. To stain the pollen, mature anthers from the inflorescence were dissected at the same developmental stage (Stage 12) and gently soaked in an Alexander staining solution for 6 h. The stamens were then placed on slides, and images were captured using an Olympus BX51 microscope (Tokyo, Japan).

### 4.9. Resin-Embedded Filament Cross-Section Assay

For semi-thin anther cross-sections, the inflorescence was dissected at the same developmental stage (Stage 13) and then fixed in 3.7% FAA, followed by dehydration through an ethanol series. After dehydration, the clearing solution (10 mL glycerol, 5 mL water, and 40 g chloral hydrate) was used for clearing, and the specimens were cleared for 12–16 h before being embedded in Spurr’s resin [27]. Cross-sections with a thickness of 2 μm were cut using the Leica RM2265 (Wetzlar, Germany). The sections were stained with 0.2% (*w*/*v*) toluidine blue in 0.1 M phosphate buffer at pH 7.0. Images were acquired using the Olympus BX51 microscope (Japan) equipped with a digital camera.

### 4.10. Accession Numbers

Sequence data from this article can be found in the *Arabidopsis* Genome Initiative database under the following accession numbers: MYB21 (AT3G27810) and KTN1 (AT1G80350).

## 5. Conclusions

Proper and precise elongation and growth of plant stamen filaments is fundamental; this complicated process determines the success of double fertilization and the continuity of a species, which is subject to strict, precise genetic control. However, the gene regulatory network that mediates filament elongation and growth in plants and the upstream signal transduction pathways remain largely unknown. In this study, we reevaluated the function of MYB21 and identified that the MYB21-KTN1 module may play a key role in fine-tuning *Arabidopsis* filament elongation.

## Data Availability

The data that support the findings of this study are available on request from the corresponding author.

## Supplementary Materials

The following supporting information can be downloaded at: https://www.mdpi.com/article/10.3390/plants12223884/s1, Figure S1: *Arabidopsis MYB21* is not involved in the regulation of filament cell number and stamen count. (A and B) The average filament cell number in the longitudinal epidermal cells (*n* ≥ 8 filaments per genotype) (A) and the stamen filament count (*n* ≥ 20 flowers per genotype) (B) were measured for Col-0, *myb21*, and the *MYB21 COM* line at the late floral stage 13. Error bars represent the standard deviation (SD) of three independent experiments. “ns” above the columns indicates no significant difference at *p* < 0.05 according to one-way ANOVA. Figure S2: The knockout mutation of *MYB21* does not result in severe developmental defects in filament vascularization. Resin-embedded filament cross-sections of Col-0, *myb21*, and the *MYB21 COM* line were examined at the floral stage 13. The red arrows indicate vascularization in the filaments. Scale bar = 20 µm. Figure S3: The loss of *MYB21* function does not result in significant defects in pollen viability in *Arabidopsis.* (A) Alexander staining reveals viable pollen grains in Col-0, *myb21*, and the *MYB21 COM* line at floral stage 12. Scale bar = 50 µm. (B) The percentage of viable pollen grains relative to the total pollen grains in the anther of Col-0, *myb21*, and the *MYB21 COM* line was quantified (*n* ≥ 6 anthers per genotype). There is no significant difference among these lines. Error bars represent the standard deviation (SD) of three independent experiments. “ns” above the columns indicates no significant difference at *p* < 0.05 according to one-way ANOVA. Table S1: The primers used in this study.
