# Supplementary material for: Arabidopsis MYB21 Negatively Regulates KTN1 to Fine-Tune the Filament Elongation
Source: Plants (Basel). 2023 Nov 17;12(22):3884. doi: 10.3390/plants12223884 (PMC10675564; doi:10.3390/plants12223884)
Supplement: Supplementary file 1 [file plants-12-03884-s001.zip › plants-2658709-supplementary.pdf]

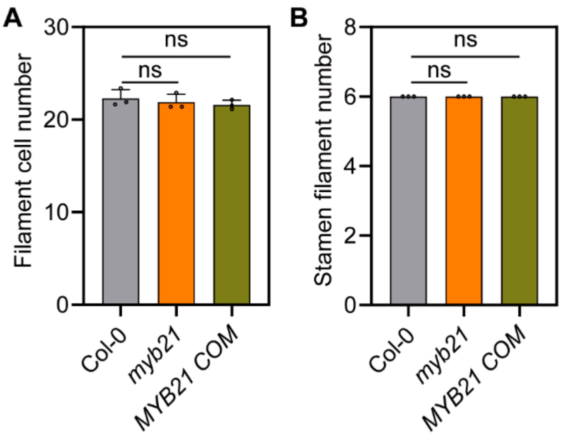

Supplemental Figure S1. *Arabidopsis MYB21* is not involved in the regulation of filament cell number and stamen count. (A and B) The average filament cell number in the longitudinal epidermal cells ($n \geq 8$ filaments per genotype) (A) and the stamen filament count ($n \geq 20$ flowers per genotype) (B) were measured for Col-0, *myb21*, and the *MYB21 COM* line at the late floral stage 13. Error bars represent the standard deviation (SD) of three independent experiments. "ns" above the columns indicates no significant difference at $P < 0.05$ according to one-way ANOVA.

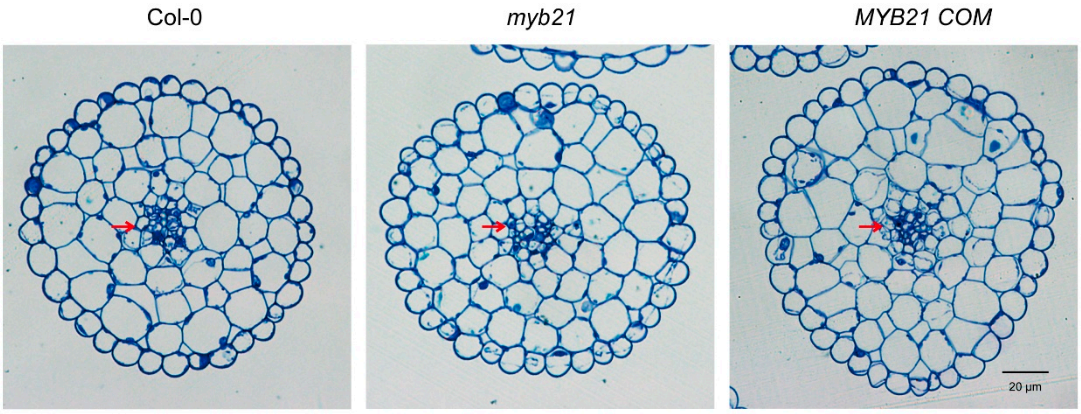

Supplemental Figure S2. The knockout mutation of *MYB21* does not result in severe developmental defects in filament vascularization. Resin-embedded filament cross-sections of Col-0, *myb21*, and the *MYB21 COM* line were examined at the floral stage 13. The red arrows indicate vascularization in the filaments. Scale bar = 20 μm.

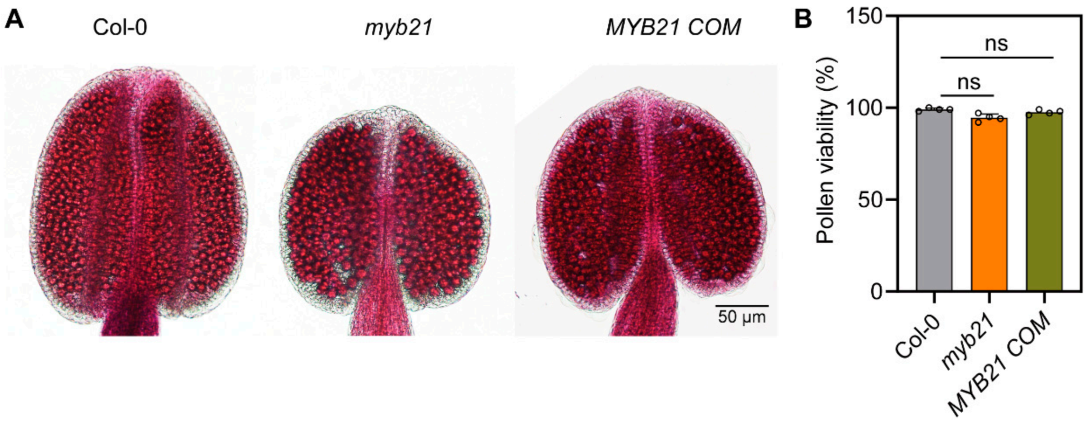

Supplemental Figure S3. The loss of *MYB21* function does not result in significant defects in pollen viability in *Arabidopsis*. (A) Alexander staining reveals viable pollen grains in Col-0, *myb21*, and the *MYB21 COM* line at floral stage 12. Scale bar = 50 µm. (B) The percentage of viable pollen grains relative to the total pollen grains in the anther of Col-0, *myb21*, and the *MYB21 COM* line was quantified ($n \geq 6$ anthers per genotype). There is no significant difference among these lines. Error bars represent the standard deviation (SD) of three independent experiments. "ns" above the columns indicates no significant difference at $P < 0.05$ according to one-way ANOVA.

Supplemental Table S1. The primers used in this study.

Primer name	primer (5’-3’)
LBb1.3	ATTTTGCCGATTTCGGAAC
myb21-LP	GCCTATTTCTCCTCCATGCTC
myb21-RP	TTTTGATCTCGTTGTCCGTTC
MYB21-LBa1	TGGTTCACGTAGTGGGCCATCG
EF1-α-qRT-F	ACGCTCTTCTTGCTTTCACC
EF1-α-qRT-R	GAGATTGGCACAAATGGGAT
KTN1-qRT-F	CCAGTGGCAATGTGTGATTTC
KTN1-qRT-R	GAGCCACTTCTCGTGTTTCT
MYB21-pB42AD-F	GTGCCAGATTATGCCATGGAGAAAAGAGGAGGAGGAAG
MYB21-pB42AD-R	CGAAGAAGTC CAAAGTCAATTACCATTCAATAAATGCA
KTN1-pro-pLacZi-F	TCGGAATTCGAGCTCAATTCCGTGACTGAGCTTGTT
KTN1-pro-pLacZi-R	AGCACATGCCTCGAGTTCCTCTTTTACTAAAAAAATAGCC
MYB21-pSuper-F	TCT AGA AAG CTT CTG ATGGAGAAAAGAGGAGGAGG
MYB21-pSuper-R	GCCCTTGCTCACCATATTACCATTCAATAAATGCATTGAT
KTN1-pro-GUS-F	TGGCTGCAGGTCGACAATTCCGTGACTGAGCTTGTT
KTN1-pro-GUS-R	GGTGGACTCCTCTTATTCCTCTTTTACTAAAAAAATAGCC
MYB21-pro-1300-F	CTGCAGTATATATGGTCACGTCAAAGTCT
MYB21-pro-1300-R	TCTAGATTTTTTTGTTATTATTCTTTATTTA
MYB21-CGFP-1300-F	TCTAGAATGGAGAAAAGAGGAGGAGGAA
MYB21-CGFP-1300-F	GGTACCTTACTTGTACAGCTCGTCCATGCCG
